# A simple PCR-based method for the rapid and accurate identification of spider mites (Tetranychidae) on cassava

**DOI:** 10.1038/s41598-020-75743-w

**Published:** 2020-11-11

**Authors:** Tatiana M. Ovalle, Aymer Andrés Vásquez-Ordóñez, Jenyfer Jimenez, Soroush Parsa, Wilmer J. Cuellar, Luis A. Becerra Lopez-Lavalle

**Affiliations:** 1 Centro Internacional de Agricultura Tropical (CIAT), Km 17, Recta Cali-Palmira, 763537 Cali, Valle del Cauca Colombia; 2 CGIAR Research Program for Root Tubers and Bananas, Lima, Peru; 3 Entomology Section, Universidad del Valle, Ciudad Universitaria Melendez, Cali, Valle del Cauca Colombia; 4 Present Address: International Potato Center (CIP), Av. La Molina 1895, La Molina, Lima, Lima12 Perú

**Keywords:** Entomology, DNA sequencing

## Abstract

The morphological identification of mites entails great challenges. Characteristics such as dorsal setae and aedeagus are widely used, but they show variations between populations, and the technique is time consuming and demands specialized taxonomic expertise that is difficult to access. A successful alternative has been to exploit a region of the mitochondrial cytochrome oxidase I (*COI*) gene to classify specimens to the species level. We analyzed the *COI* sequences of four mite species associated with cassava and classified them definitively by detailed morphological examinations. We then developed an identification kit based on the restriction fragment length polymorphism–polymerase chain reaction of subunit I of the *COI* gene focused on the three restriction enzymes *Ase*I, *Mbo*II, and *Apo*I. This set of enzymes permitted the simple, accurate identification of *Mononychellus caribbeanae*, *M. tanajoa*, *M. mcgregori*, *and Tetranychus urticae*, rapidly and with few resources*.* This kit could be a vital tool for the surveillance and monitoring of mite pests in cassava crop protection programs in Africa, Asia, and Latin America.

## Introduction

Cassava (*Manihot esculenta* Crantz) performs well in poor soils, even in regions with unpredictable rainfall, where the cultivation of cereals and other crops is challenging or impossible. Ecology and climate models suggest that even though cassava should continue to produce well under extreme climatic shifts, it may become increasingly subject to the geographic shift of pests and diseases^1,2^. Models predicting pest incidence suggest that, due to climate change, the distribution range of mites will widen to cover almost all areas where cassava has the chance to predominate^1^.

More than 50 species of phytophagous mites are associated with cassava; among those, the spider mites (*Tetranychus urticae*, *T. cinnabarinus*, *Mononychellus caribbeanae*, *M. tanajoa*, and *M. progressivus*) cause the most damage, decreasing root yield by 87% and the number of stem cuttings in 82% of plants^3^. Recently, there have been reports of mites in Asia causing significant yield reductions: 2–10% in the case of slight damage and up to 60% of serious damage^4,5^. Moreover, in the last decade, *Mononychellus mcgregori* has been reported in new cassava cropping areas in Vietnam and Cambodia^6,7^, raising concerns with farmers of its potential spread into new fields. The vegetative propagation of crops like cassava tends to cause a build-up of pathogens and pest infestations, which makes disease diagnosis difficult. Symptoms caused by different pests and diseases (e.g., mites, mosaic viruses, and mealybugs) are found commonly in the field, making pest surveillance and monitoring cumbersome. In Africa, the 1970s outbreak of cassava green mites confounded the severity of both cassava mosaic disease and the mealybug, making it very difficult to implement timely control and management measures^8^. Therefore, an integrated approach to monitor and manage these pests must include a reliable and easy species-identification assay.

Nowadays, molecular biological methods are used widely for species identification in molecular entomology. DNA barcoding using the ∼650-bp region near the 5′-end of the mitochondrial cytochrome c oxidase subunit I (*COI*) gene^9^ is a useful and reliable species-identification marker in insects, due to its lack of introns, limited exposure to recombination, and the availability of robust primer sites. *COI* has been employed effectively for species-level discrimination in insects^10–12^ and arachnid^13^. In this study, our objectives were to develop an identification kit based on restriction fragment length polymorphism–polymerase chain reaction (RFLP-PCR) using subunit I of *COI* to rapidly and definitively identify four species of cassava mites, and to democratize its access to field practitioners to ameliorate the current labor-intensive and time-consuming taxonomic identification methodology.

## Materials and methods

### Establishment of mite colonies

Cassava leaves with mites and mite damage were collected from four locations at Valle del Cauca, Colombia: a producer field located at the locality of Potrerillo (municipality of Palmira); an experimental field; and two greenhouses at the International Center for Tropical Agriculture, Palmira (CIAT, from its Spanish acronym) (Table 1). At each location, at least 30 males and 30 females of the same morphotype were collected under a stereomicroscope and placed on one-month-old cassava plants for rearing. Each colony was checked every three days until teliochrysalids, the last developmental stage before adult emergence, were detected. From each colony, one to three couples of newly emerged females (virgin) and non-virgin males were placed on a new cassava plant in a rearing room (25 °C and 70% relative humidity) at the CIAT (Table 1) to establish a new colony from these founders. The new colonies were maintained until at least a hundred adult mites were detected; at this point, the leaves with mites were collected and placed into a freezer at − 20 °C for several minutes. Dead mites were collected and placed into an empty vial (Eppendorf Tubes™, Germany). For each colony, 13–28 adults of both sexes were mounted for morphological classification; the remaining individuals were preserved at − 80 °C and used for molecular studies.

**Table 1:** Data on the four mite colonies raised on cassava.

Colony number	Number of founding couples^a^	Scientific names^b^	Location^c^	Geographic coordinates^c^	Collection date^c^
1	2	*Mononychellus caribbeanae* (17/11)	CIAT (Greenhouse)	3.501151°N, 76.357478°W	July 2012
2	3	*M. mcgregori* (12/4)	Alto del Tigre, El Olivo, Potrerillo (Field)	3.546583°N, 76.179129°W	February 2012
3	4	*M. tanajoa* (8/7)	CIAT (Field)	3.500928°N, 76.350930°W	July 2012
4	2	*Tetranychus urticae* (17/1)	CIAT (Greenhouse)	3.499676°N, 76.358534°W	July 9, 2012

^a^This number corresponds to the founding couples selected for the formation of a new colony.

^b^The morphological identifications were made by Aymer Andrés Vásquez-Ordóñez (the CIAT, see the “Morphological identification” section). The number of females and males, respectively, examined for identification is indicated in parentheses.

^c^The location, geographic coordinates, and collection date correspond to data for the collection of mites used to establish the first colonies. The mites collected in the greenhouses were wild mites, not cultured mites.

### Morphological identification

Seventy-seven adults from the four colonies indicated in Table 1 were cleared in lactophenol solution and mounted on slides in Hoyer’s solution, following the guidelines of Krantz and Walter^14^. Each specimen was examined, and the most relevant morphological structures for identification were photographed with scales using a Canon Eos 60D camera attached to an Olympus light microscope BX43, and with a Nikon Digital Sight DS-Ri1 camera attached to a Nikon Eclipse Ni-U 90 microscope. The measurements (micrometers μm) were developed in Adobe Illustrator (Adobe Systems Incorporated, USA). The body length represented by the idiosoma, was measured with and without gnathosoma, and the setae were measured from the base to the tip; the description of characters follows Lindquist^15^. Identification to the genus level was performed using the taxonomic key of Bolland et al.^16^, and the species were identified with different taxonomic keys^17^, morphological descriptions, illustrations^18–20^ and 55 specimens identified by José Maria Guerrero at CIAT, for each genus identified. All specimens examined were deposited in the CIAT Arthropod Reference Collection (CIAT-ARC).

### DNA isolation and PCR amplification

DNA was extracted from four mite species using the cetyltrimethylammonium bromide method^21^, with slight modifications (potassium acetate 2.5 M, pH 5.5). The DNA barcode region was amplified using 10 μM of universal DNA primers LCO1490 (5′-GGTCAACAAATCATAAAGATATTGG-3′) and HCO2198 (5′-TAAACTTCAGGGTGACCAAAAAATCA-3′) for the amplification of *COI*^22^. PCR was performed in a 20-µl reaction volume using 0.25 U of Platinum® Taq (Invitrogen®, USA), 1X PCR buffer [200 mM Tris HCl (pH 8.4), 500 mM KCl], 2.5 mM of each dNTP (Promega Corp., USA), 2.5 mM MgCl_2_, and 20 ng DNA. The amplification program, conducted in a Mastercycler® Pro (Eppendorf, Germany), was 5 cycles (40 s at 94 °C, 40 s at 45 °C, and 60 s at 72 °C), followed by 35 cycles (40 s at 94 °C, 40 s at 51 °C, and 60 s at 72 °C) with a final extension period of 72 °C for 10 min.

### Sequencing of the *COI* region

The *COI* region of each green mite species was cloned into the PGEM®-T Easy vector (Promega Corp., USA). Plasmid DNA from *Escherichia coli* was purified using an SV minipreps preparation (Promega Corp., USA). DNA inserts in this vector were sequenced in both directions using the Big Dye™ Terminator Cycle Sequencing kit with an Applied Biosystems 377 DNA fragment analyzer by the Cornell University Life Sciences Core Laboratories Center (Ithaca, NY, USA) using vector primers SP6 and T7. The resulting DNA sequences were edited and analyzed using Sequencher® 4.5 (Gene Codes Corp., USA).

### DNA sequence analysis

DNA sequences were edited by eliminating vector and universal primer sequences with Sequencher V5.2.4. *COI* sequence contigs were assembled from three clones per species to identify unique haplotypes with a discrimination requirement of 100% identity. All mite sequence identities and similarities were calculated using BLAST. Genetic distances were calculated with the alignment sequences for the four species. Additionally, we included *COI* sequences reported in GenBank from other species belonging to the same tribe (Tetranychini) (Table 2). The alignment process was carried out using both Mezquite and MEGA v7.0 software^23^. The phylogenetic reconstruction analyses were performed using the parameters from the Neighbor-Joining model^24^ and Kimura-2 model^25^ for nucleotides (nt) with 1000 bootstrap replications.

**Table 2:** List of mite species and their *COI* regions published in GenBank.

Species	Tribe	GenBank ID
*Amphitetranychus viennensis*	Tetranychini	KC136028.1
*Oligonychus perseae*	Tetranychini	KF011470.1
*Oligonychus punicae*	Tetranychini	KF011453.1
*Panonychus citri*	Tetranychini	KC136099.1
*Panonychus ulmi*	Tetranychini	NC_012571.1
*Stigmaeopsis longus*	Tetranychini	AB531835.1
*Stigmaeopsis miscanthi*	Tetranychini	AB429422.2
*Tetranychus cinnabarinus*	Tetranychini	HM753535.1
*Tetranychus evansi*	Tetranychini	KX281695.1
*Tetranychus kanzawai*	Tetranychini	NC 024676.1
*Tetranychus ludeni*	Tetranychini	KX281694.1
*Tetranychus malaysiensis*	Tetranychini	NC_024678.1
*Tetranychus pueraricola*	Tetranychini	MG518353.1
*Tetranychus truncates*	Tetranychini	MG518331.1
*Tetranychus urticae*	Tetranychini	KC136132.1
*Bryobia sp.*	Bryobiini	HQ991528.1

### Restriction mapping and *COI*-RFLP assay

*COI* sequences belonging to each of the four mite species morphologically identified in this study, and the 15 Tetranychini and 1 *Bryobia COI* sequences obtained from GenBank for the species identified as closely related to them (Table 2), were digested in silico through the web portal NEBcutter V2.0^26^. In total, 36 restriction enzymes were evaluated to estimate whether the *COI* region’s restriction pattern could be used to identify the four mite pest species associated with cassava and to select the most informative restriction pattern and its enzyme(s). Of the 36 commercially available restriction enzymes, three allowed a clear differentiation of the four mite species in silico. Two units of the restriction enzymes *Ase*I, *Mb*oII, and *Apo*I were used to digest 2 µg of the PCR-amplified *COI* region from *T. urticae*, *M. caribbeanae*, *M. mcgregori*, and *M. tanajoa.* The final reaction volume was 50 µl and included 10% of 1X supplied buffer and ultrapure water. Reactions were incubated for 1 h at 37 °C or 50 °C according to the manufacturer’s specifications. Restriction digestion products were run in 2% agarose gel in BS buffer (10% boric acid, 2% sodium hydroxide) at 80 V for 3 h. Gel staining was carried out with ready-made SyBR Safe (Invitrogen, USA). Restriction pattern images were captured with a GelDoc™ BioRad documentation system, and size estimation of the digestion patterns was performed using PyElph V.1.4 software^27^.

## Results

### Morphological identification

In the Valle del Cauca region of Colombia, cassava is affected by different mite species. A survey conducted in 2012, in which cassava leaves infested by mites were collected and inspected under a stereoscope, identified at least four morphotypes on a single leaf. Four mite colonies, one from each morphotype, were then established on fresh cassava leaves. From the resulting offspring, mating couples were selected and allowed to breed on new cassava leaves to enable a full taxonomic and molecular identification (Table 1). A complete morphological characterization was undertaken on each mite colony, allowing the identification of four species: *Mononychellus caribbeanae*^28^, *M. mcgregori*^29^, *M.* *tanajoa*^30^, and *Tetranychus urticae*^31^. A description of the diagnostic morphological characteristics used to identify these species is provided in detail.

### Taxonomy

Family Tetranychidae Donnadieu.

Subfamily Tetranychinae Berlese, 1913.

Tribe Tetranychini Reck, 1950.

Genus *Mononychellus* Wainstein, 1971.

*Diagnosis* Two pairs of para-anal setae (h2-3). Empodium split near the middle into three pairs of hairs. Opisthosoma with longitudinal striae between the e1 setae. Dorsal body setae serrate^16^.

#### *Mononychellus caribbeanae* McGregor, 1950^28^

##### Diagnosis

**Figure 1:**
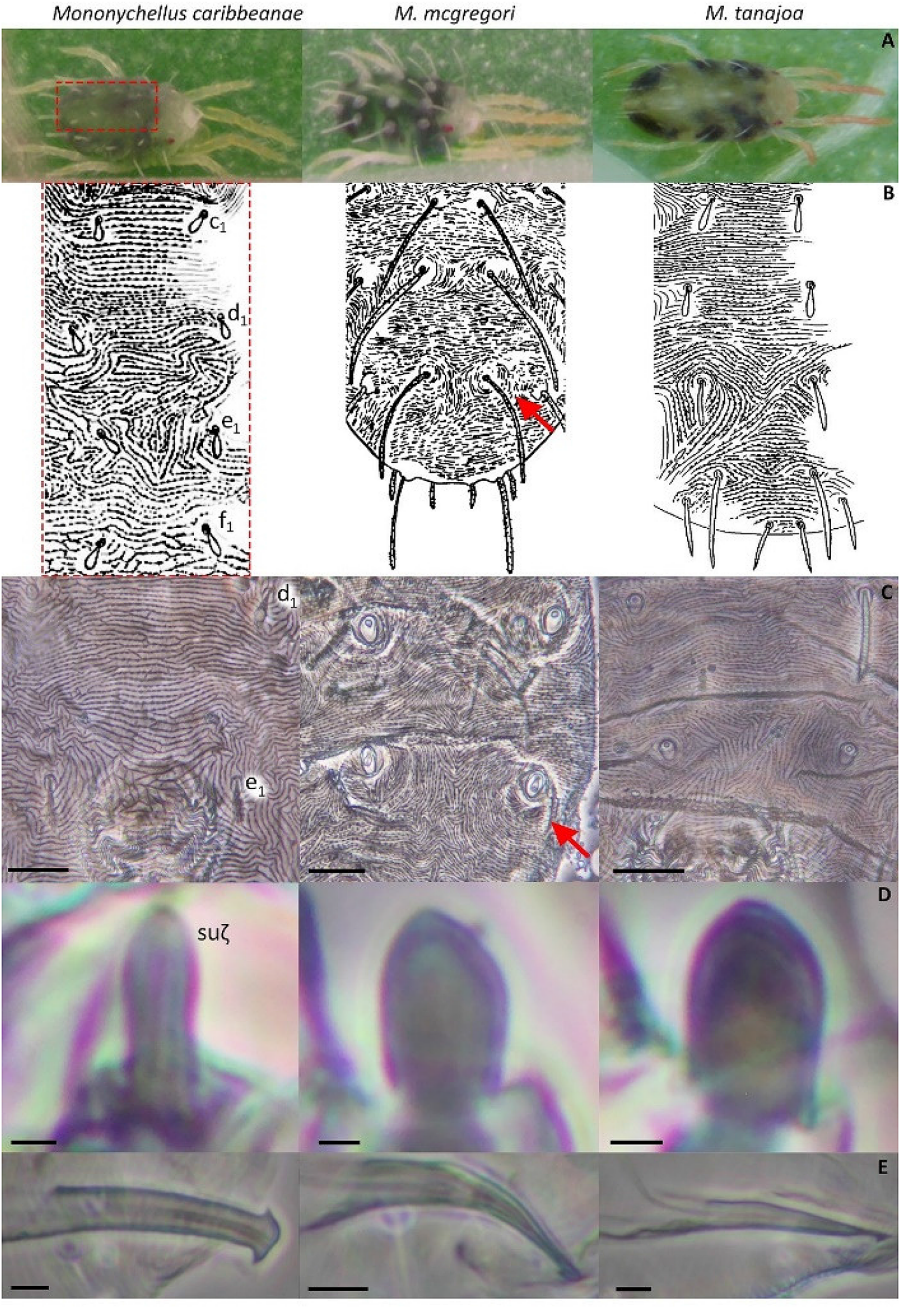
Morphological diagnostics characters for *Mononychellus caribbeanae* (left), *M. mcgregori* (center) and *M. tanajoa* (right) collected in the Valle del Cauca region. Habitus on plants (**A**); hysterosoma in dorsal view between setae c1–f1 (**B**, red rectangle) and approach to the area between the setae d1–e1 (**C**), tubercles indicated with a red arrow; palptarsus, terminal eupathidium (D, indicated as suζ); terminal aedagus of males (**E**). (**C**–**E**) with scales bar of 20 and 2 µm, respectively. Figures B from Tuttle et al. (1976, 1977)^19,20^ and Flechtmann (1982)^18^. Pictures by Rodrigo Zuñiga and Aymer Andrés Vásquez-Ordóñez.

Female: Length of body with gnathosona of 520 (429–455) and without gnathosoma of 422 (507–533), width of 344 (325–364) (Fig. 1A)^18^. Dorsal striae of hysterosoma anastomosed except in the posterior part (caudally to f1 setae) where there is a reticulate pattern (Fig. 1B,C)^17,18,32^. Dorsal body setae pubescent, clavate, and not on tubercles (Fig. 1B)^32^. The setae c1, d1, e1 and f1 are less than half the distance between their bases (Fig. 1B)^18^. Terminal eupathidium on palptarsus 1.0 to 2.9 as long as broad (Fig. 1D)^17,33,34^. Male: Aedeagus curving slightly on dorsal margin; knob with anterior margin convex and with margin dorsal and ventral on acute angles approximately equal (Fig. 1E)^35^.

##### Materials examined

Twenty-seven females and eight males, Cesar (n = 3), La Guajira (1) and Valle del Cauca (22), Colombia; two females, Cocle, Panama; two females, Managas and Sucre, Venezuela; one female, Palmar del Rio, Cuba. All specimens were collected on *Manihot esculenta* (Euphorbiaceae).

#### *Mononychellus mcgregori* Flechtmann & Baker, 1970^29^

##### Diagnosis

**Figure 2:**
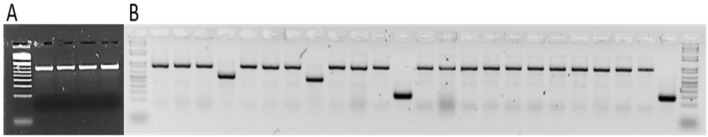
(**A**) *COI* region amplification in mite species. (**B**) Confirmation of the presence of the fragment of interest in the *E. coli* genome using the universal primers T7 and SP6.

Female: Length of body with gnathosona of 424 (403–442) and without gnathosoma of 364 (351–377), width of 281 (260–299) (Fig. 1A)^18^. Dorsal striae of hysterosoma transverse (Fig. 1B,C)^18,29^. Dorsal body setae long and serrate, on tubercles (Fig. 1B)^18,29^. The setae c1, d1, e1 and f1 longer than the distance between their bases (Fig. 1B)^17,18^. Terminal eupathidium on palptarsus 1.0 to 1.9 as long as broad (Fig. 1D, Fig. 2). Male: Aedeagus bent ventral; knob small, triangular, with posterior margin rounded and anterior one sharp-pointed (Fig. 1E)^35^.

##### Materials examined

Eleven females and four males, Cauca (1), Huila (1), Quindio (1) and Valle del Cauca (8), Colombia; two females, Junin, Peru; two females, Quan Ngai, Vietnam. All specimens were collected on *Manihot esculenta* (Euphorbiaceae).

#### *Mononychellus tanajoa* Bondar, 1938^30^

##### Diagnosis

Female: Length of body with gnathosona of 458 (431–481) and without gnathosoma of 398 (360–481), width of 281 (273–299) (Fig. 1A)^18^. Dorsal striae of hysterosoma form a slightly reticulate pattern and transverse (Fig. 1B,C). Dorsal body setae not on tubercles and setae c1, d1, e1 and f1 are less than half or two thirds than the distance between their bases (Fig. 1B)^17,18,36^. Terminal eupathidium on palptarsus 1.0 to 2.1 as long as broad (Figs. 1D, 2)^17^. Male: Aedeagus straight turns ventral apically at a slight angle and with distal angulations (Fig. 1E)^18,20,36,37^.

##### Materials examined

Forty-one females and seventeen males, Atlántico (1), Guajira (1), Magdalena (2), Meta (1) and Valle del Cauca (36), Colombia; seven females, Anzoategui (1), Apure (1), Carabobo (1) and Monagas (3), Venezuela; two females, Idiho and Fonkome, Benin. All specimens were collected on *Manihot esculenta* (Euphorbiaceae).

#### Genus *Tetranychus* Dufour

##### Diagnosis

One pair of para-anal setae. Empodium split distally, usually into three pairs of hairs, and spur absent. Duplex setae of tarsus I well separated. Peritreme anastomosed distally^16^.

#### *Tetranychus urticae* Koch, 1836^31^

##### Diagnosis

**Figure 3:**
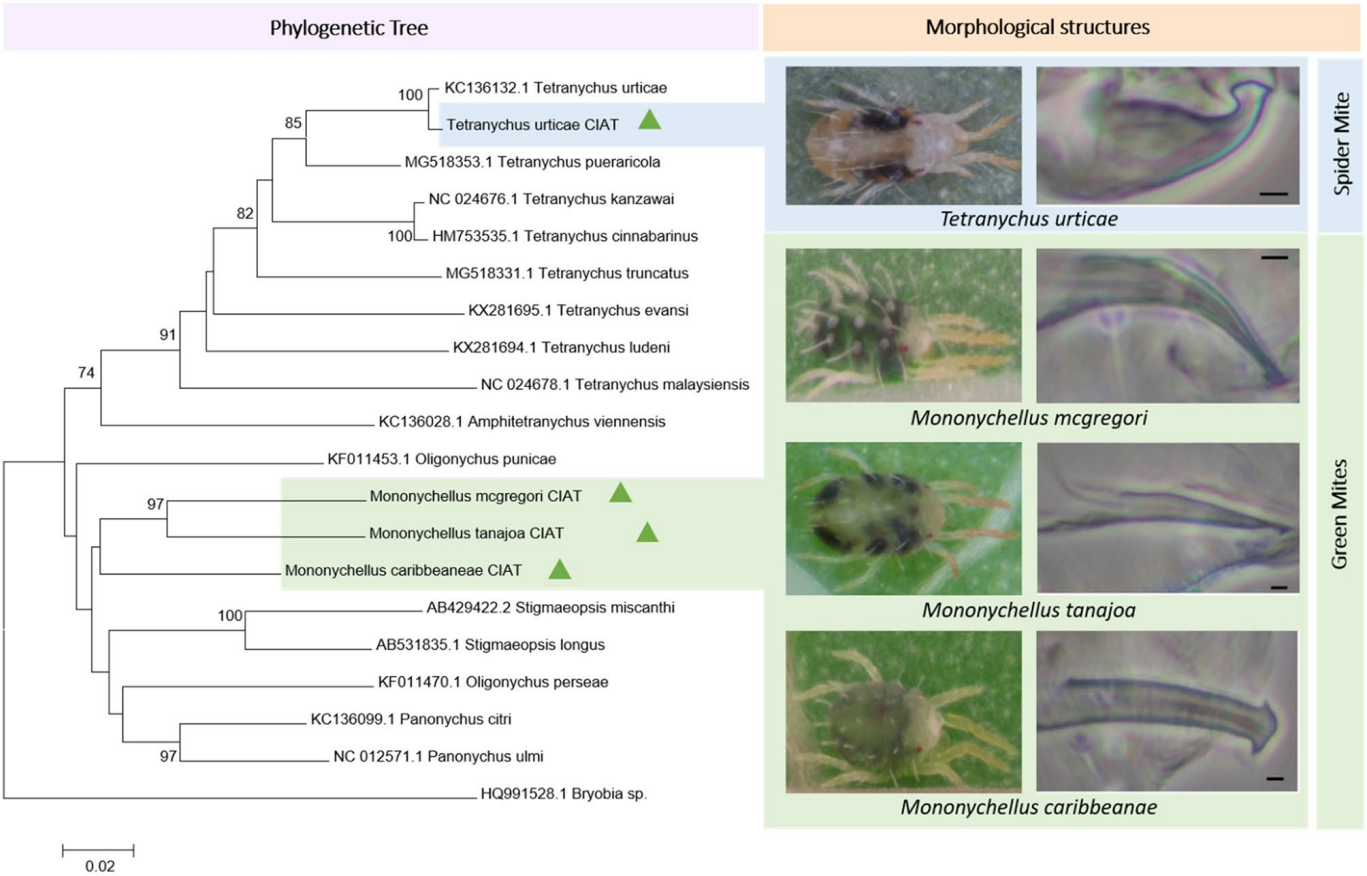
Left panel: Phylogenetic tree depicting the relationships between the mites. The tree was based on nucleotide sequences from the *COI* gene and generated using MEGA v7.0^23^, with the Neighbor-Joining method and with distances calculated using the Kimura-2 parameter. Right panel: Images of mite species collected in the Valle del Cauca region and morphological structures to identify the male aedeagus. Scale bars = 2 µm. Pictures by Rodrigo Zuñiga and Aymer Andrés Vásquez-Ordóñez (CIAT).

Female: Length of body with gnathosona of 500^38^, with elliptical body shape (Fig. 3). Empodia with three pairs of proximoventral hairs and without dorsomedian spur. Peritremes with a hook longer than 15. Dorsohysterosomal striae longitudinal between members of setae e1 and members of setae f1, forming a diamond shaped pattern. Tarsus I with the socket of proximal duplex setae distal (> 10) to the socket of four proximal tactile setae. Preginital striae broken medially and solid laterally. Male: Empodium of leg I-II with mediodorsal spurs longer than 2 µm. Terminal knob of aedeagus less than twice (about 1.5x) as wide as neck and parallel or forming a small angle (0–20°) with axis of shaft; knob rounded or angulated dorsally and with anterior and posterior acute projection (Fig. 3)^39–41^.

##### Materials examined

Fifty-six females and nine males from International Center for Tropical Agriculture, Palmira, Colombia (3.499754, -76.353972), on *Manihot esculenta*.

### Key of cassava spider mite species of this study

Note: Based and modified of Bolland et al.^16^ and Flechtmann & de Queiroz^17^.

1. Female and male: One pair of para-anal setae…*Tetranychus urticae*.

Female and male: Two pairs of para-anal setae (h2-3)…2.

2. Female: Dorsal striae of hysterosoma anastomosed except in the posterior part (caudally to f1 setae) where there is a reticulate pattern (Fig. 1B,C). Male: Knob of aedeagus with anterior margin convex and with margin dorsal and ventral on acute angles approximately equal (Fig. 1E)…*Mononychellus caribbeanae*.

Female: Dorsal striae of hysterosoma form a transverse or slightly reticulate pattern and transverse (Fig. 1B,C). Male: Knob of aedeagus small, triangular, and with a distal sharp-pointed or angulations (Fig. 1E)…3.

3. Female: Dorsal striae of hysterosoma transverse (Fig. 1B,C). Dorsal body setae on tubercles (Fig. 1A–C). The setae c1, d1, e1 and f1 longer than the distance between their bases (Fig. 1A,B). Male: Aedeagus bent ventral; knob small, triangular, with posterior margin rounded and anterior one sharp-pointed (Fig. 1E)…*Mononychellus mcgregori*.

Female: Dorsal striae of hysterosoma form a slightly reticulate pattern and transverse (Fig. 1B,C). Dorsal body setae not on tubercles (Fig. 1A–C). The setae c1, d1, e1 and f1 are less than half or two thirds than the distance between their bases (Fig. 1A,B). Male: Aedeagus straight turns ventral apically at a slight angle and with distal angulations (Fig. 1E)…*Mononychellus tanajoa*.

### Sequencing and phylogenetic analysis

Standard PCR was performed using the *COI*-barcode primers on DNA extracted from the four mite species. A positive amplification of the *COI* region was resolved in 1.5% agarose (Fig. 2A). The *COI* region of each sample was then cloned, and positive clones containing an insert of the relevant size were confirmed by fragment amplification using T7 and SP6 primers (Fig. 2B). A total of 12 samples, three individual clones per species, were sent for sequencing. The length of the DNA sequences obtained was 709 bp, with a unique sequence haplotype for each mite species. Sequence analysis confirmed the presence of the 219 amino acids of the COX1 gene present in the mitochondrial genome and commonly referred to as the *COI* sequence or the Barcode of Life^42^.

A BLAST search of the four mite haplotype sequences against the NCBI’s GenBank revealed six mite genera closely related to these samples. Thus, we proposed a global alignment that included 16 *COI* DNA sequences plus the four haplotype sequences from this study; this alignment was used in a phylogenetic reconstruction.

A phylogenetic tree was constructed based on the nucleotide sequence alignment, with bootstrap values above 70% based on 1000 replicates; these values are indicated at each node (Fig. 3). Most of the nodes in the tree were highly supported. An example species from the genus *Bryobia* was used as an outgroup.

This phylogenetic tree clearly separates the genus *Tetranychus* (*T. urticae*) from the genus *Mononychellus* (*M. tanajoa*, *M. mcgregori*, and *M. caribbeanae*). The *T. urticae* example from this study clusters together with the GenBank example of the same species collected in Korea. Likewise, the three species of *Mononychellus* collected in the Valle del Cauca clustered together; they are the first to be sequenced and reported to GenBank.

### COI-RFLP analysis

**Figure 4:**
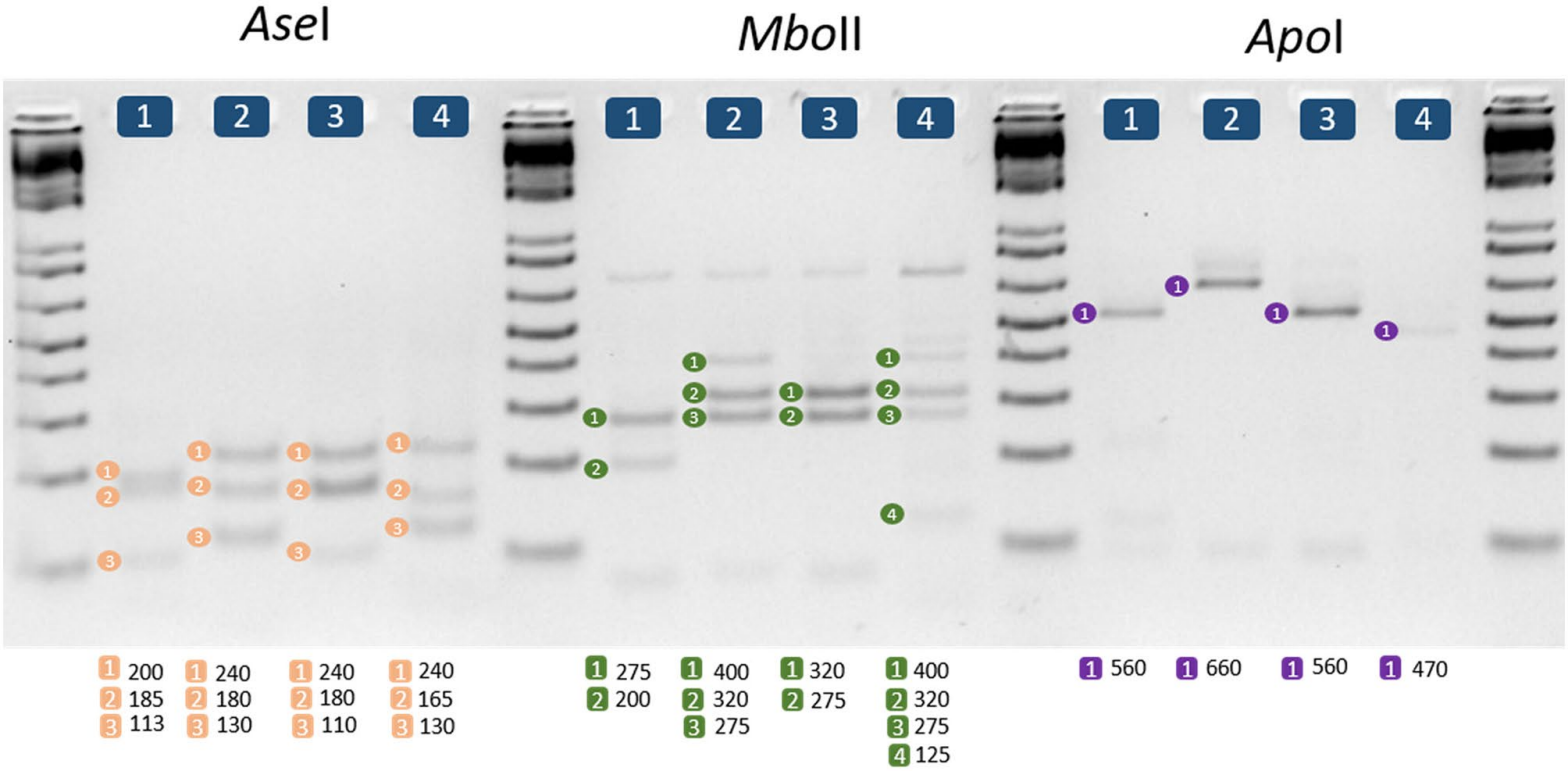
Agarose gel electrophoresis restriction patterns for the enzymes *Ase*I, *Mbo*II, and *Apo*I in the four mite species. Lane 1, *Mononychellus caribbeanae*; lane 2, *Mononychellus mcgregori*; lane 3, *Mononychellus tanajoa*; and lane 4, *Tetranychus urticae*. Molecular size markers (1 kb plus, Invitrogen) are shown on the right and left of each restriction enzyme digestion pattern. The columns of numbers below the images correspond to the fragment sizes needed to definitively identify each mite species.

*COI* sequences belonging to 20 mite species were digested in silico with 36 restriction enzymes with the aim of identifying a set of enzymes that could be used for easy identification. We found that the digestion patterns obtained with *Ase*I, *Mbo*II, and *Apo*I allowed for the full identification of the four mite species described here. Restriction digestion of the PCR-amplified *COI* regions of the four mite species confirmed the in silico results. The banding patterns obtained from the agarose gel electrophoresis showed that *Ase*I and *Mbo*II resolved eight and five distinctive bands, respectively, across all mite species with sizes above 100 bp, demonstrating the ease and accuracy of this method. *Apo*I showed only three distinctive bands, and did not distinguish between *M. tanajoa* and *M. caribbeanae*, limiting its potential use for quick diagnosis (Fig. 4).

The banding patterns obtained after restriction enzyme digestion of the *COI* region could be replicated in two independent molecular laboratories of the cassava program at the CIAT (the Genetics and the Virology laboratories), confirming the reproducibility and accuracy of RFLP-PCR. *Ase*I showed the best profile for differentiating among the four mite species, followed by *Mbo*II*.* Even though *Apo*I only allowed the definitive identification of two mite species, its inclusion alongside the other two enzymes would make an identification kit for the four species more robust.

## Discussion

In the present study, we undertook a classical morphological identification of four mite species associated with the cassava crop in Valle del Cauca and then used those results to confirm the feasibility of a proposed molecular kit for easier identification.

The morphological identification of the four mite species assessed here was challenging because it required extensive technical expertise to prepare and mount the samples as well as a deep knowledge of the taxonomy of mites. Rogo et al.^43^ and Guerrero et al.^37^ reported problems in the identification of *Mononychellus* specimens, whereby apparent differences in morphological characteristics could have been the result of bad mounting or deterioration of the microscope slide. The other limiting factor affecting the morphological characterization of *M*o*nonychellus* spp. is the limited number of morphological characteristics available for accurate characterization. For instance, in *M. tanajoa*, the traditional characteristic is the longitude of dorsal setae that presents a continuous variation among the populations, a factor that does not facilitate distinguishing them from nearby species^37,43^. Another important characteristic is the shape of the aedeagus; although its descriptions have presented problems, comprehensive research indicates the same form as documented in this paper^37,43^, demonstrating its usefulness.

Not only is the identification process for mites using morphological traits time consuming and demanding of specialized taxonomic expertise, but the task is also amplified by the fact that the Tetranychidae family is composed of > 1200 species^16^. The organism’s small size and significant phenotypic plasticity also increase the complexity associated with its morphological identification^44^.

DNA barcoding could circumvent these limitations^45^. The *COI* gene sequence has been used extensively for insect identification; for instance, it has been used successfully in the identification of whitefly^12,46^, rhopalids^47^, and mirids^48^. On this basis, we sought a method to reduce the time and increase the accuracy of mite species identification that would be usable by other scientists or agriculture research practitioners responsible for pest surveillance and monitoring, choosing to characterize the genetic information contained in the *COI* region of the COX-1 gene of four mite species taxonomically identified and associated with the cassava crop in the Valle del Cauca, Colombia.

The *COI* region has been used successfully to characterize the mite species complex^49–51^; thus, we believed this type of data would aid the species identification of mites associated with cassava. Among the most important species, only a partial *COI* sequence for *M. progressivus* is available at NCBI^52^; thus, the four haplotype *COI* sequences reported here constitute the first to be available through GenBank for the genera *Mononychellus* and *Tetranychus*. Comparing molecular markers with diagnostic morphological traits allows the building of a species-specific sequence library to aid the accurate identification in the absence of a well-trained entomologist. *COI* sequences from mites associated with other plants, including *T. urticae*^44,53–55^, were used to conduct a phylogenetic relationship analysis, permitting the construction of a phylogenetic tree from 20 *COI* sequences, including the outgroup *Bryobia* sp.^44^. Two well-defined clades were apparent: one from a monophyletic group that corresponded to the *Tetranychus* genus (and in which our *T. urticae* sample was grouped in close relationship with a sample reported by the Korea Research Institute of Bioscience and Biotechnology); and the second from a polyphyletic group composed of four different genera but including a well-defined *Mononychellus* clade.

Most mite identification studies agree on the challenge of identifying the species by morphology alone, because that is time consuming and defeats any attempt to rapidly provide information on pest distribution. Large-scale surveys involving DNA sequencing may be possible, but are probably too costly. Hence, DNA-based methods not involving sequencing become attractive. Restriction enzyme-mediated genotyping is a more cost-effective approach for identification with large sample sizes. It requires that a restriction digestion of a PCR product (RFLP-PCR) be obtained from a variable well-known gene, such as the *COI* sequence. We have shown that a standard PCR amplification of the *COI* region, followed by enzymatic digestion with *Ase*I or *Mbo*II allows the unequivocal identification of all four mite species associated with the cassava crop, with no sequencing of the *COI* region necessary. Our method does not compare sequences to classify one sample into a species; therefore, our method does not take into account the number of intra- or interspecific variations, which have been problematic, given that in some species of mite, intraspecific variation may exceed interspecific variation^44^. Our kit is based on the target sequences of the restriction enzymes and the patterns they generate when digesting the sequence of the *COI* region. This approach to a rapid and accurate mite species identification is cost effective and easy to implement in a very basic molecular biology laboratory. Thus, incorporating RFLP-PCR for the *COI* gene into a routine mite surveillance and monitoring program should quickly and simply identify any potential outbreak of an exotic type not reported previously. The restriction digestion patterns from *Ase*I and *Mbo*II clearly showed how the *COI*-RFLP is as effective a method for mite identification as that proposed for whiteflies and the oriental fruit fly^12,56^.

In conclusion, our *COI*-based RFLP-PCR kit, developed using a multi-disciplinary approach that included gene data, morphological traits, and bioinformatics pipelines, has been shown to unequivocally identify *T. urticae*, *M. caribbeanae*, *M. mcgregori*, and *M. tanajoa*. This kit can identify mite species accurately, cheaply, and rapidly, and could become a useful tool in crop protection programs monitoring and surveying mite pests.
